# Supplementary material for: Copper-Electroplating-Modified Liquid Metal Microfluidic Electrodes
Source: Sensors (Basel). 2022 Feb 25;22(5):1820. doi: 10.3390/s22051820 (PMC8915017; doi:10.3390/s22051820)
Supplement: Supplementary file 1 [file sensors-22-01820-s001.zip › Supplementary Figures.pdf]

sensors

MDPI

Supplementary Materials

Chip fabrication

First, standard soft lithography technology was used to manufacture the microchannel pattern of the side-wall micro-electroplated chip, as shown in figure S1(a). SU-8 2050 was spin-coated on a silicon wafer. After a series of steps including exposure, development, and hard bake, the raised microchannel patterns were formed by solidified SU8 on the silicon wafer. Second, the prepared PDMS was used for transferring patterns. Then the chip was sealed by plasma bonding (figure S1(b)). Third, as shown in figure S1(c), pure Gallium was heated to melt and injected into the microchannels to form the microelectrodes. The 705 glue was used to fix the wires which were connected with the microelectrode holes.

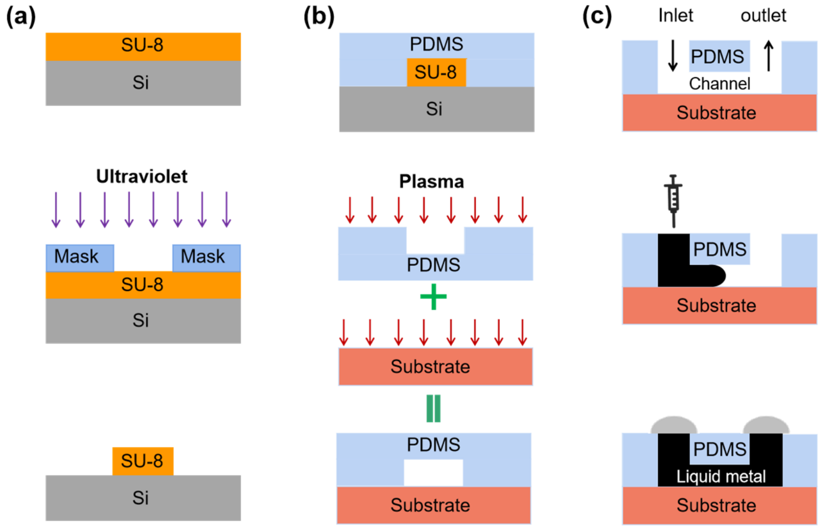

Figure S1. The process of chip fabrication. (**a**) Soft lithography process. (**b**) PDMS transfer and plasma bonding. (**c**) Injection of liquid metal to form microelectrodes.

Physical map of side-wall micro-electroplated chip

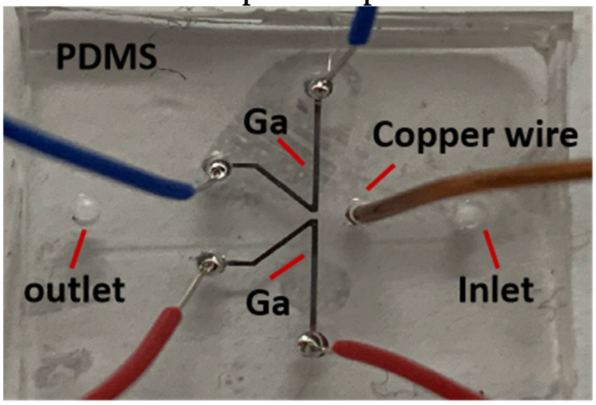

Figure S2. Physical map of side-wall micro-electroplated chip.

Copper layer growth records (observed by the microscope)

In order to draw a copper layer growth curve, the microscope was used to observe the condition of copper layer growth at different voltages for different microchips, and recorded by photographs for every 30 s, which is shown in figure S3.

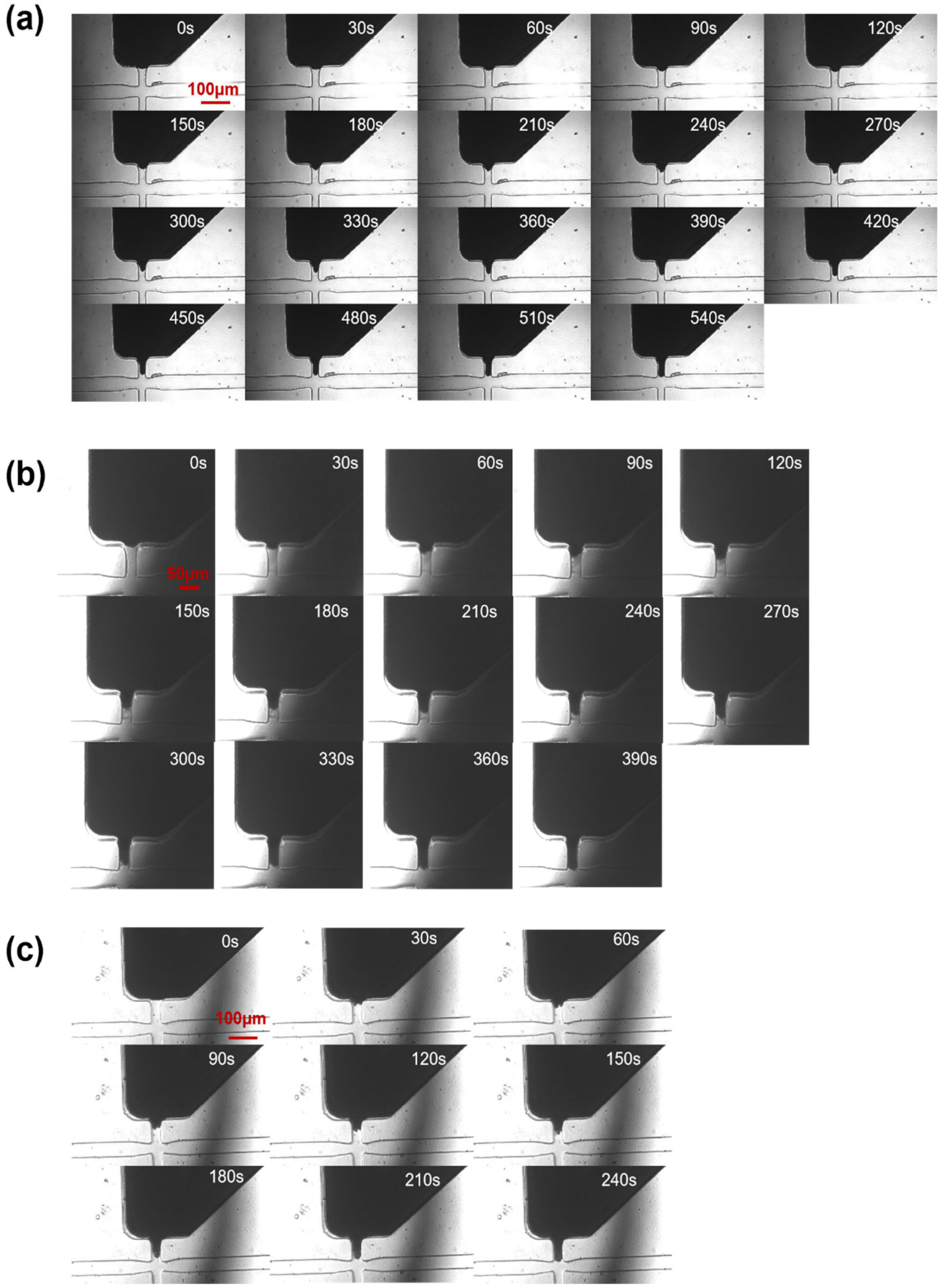

Figure S3. Copper layer growth records every 30 s at different voltages. (**a**) The copper layer growth record at 0.6 V (Time required: 540s). (**b**) The copper layer growth record at 0.8 V (Time required: 390 s). (**c**) The copper layer growth record at 1.0 V (Time required: 240 s).

Comparative experiment for metal particle counting in oil

To verify that the liquid metal microelectrodes after electroplating has better sensitivity of micro-capacitance detection than the non-electroplated ones, the non-electroplated microelectrodes were also used for counting metal particles in the oil. Figure S4 shows the result of the experiments, it could be seen that no capacitance signal was

generated when metal particles passed through due to too much spacing between the microelectrodes. In this case, noise values produced by the LCR metal were greater than the signal values.

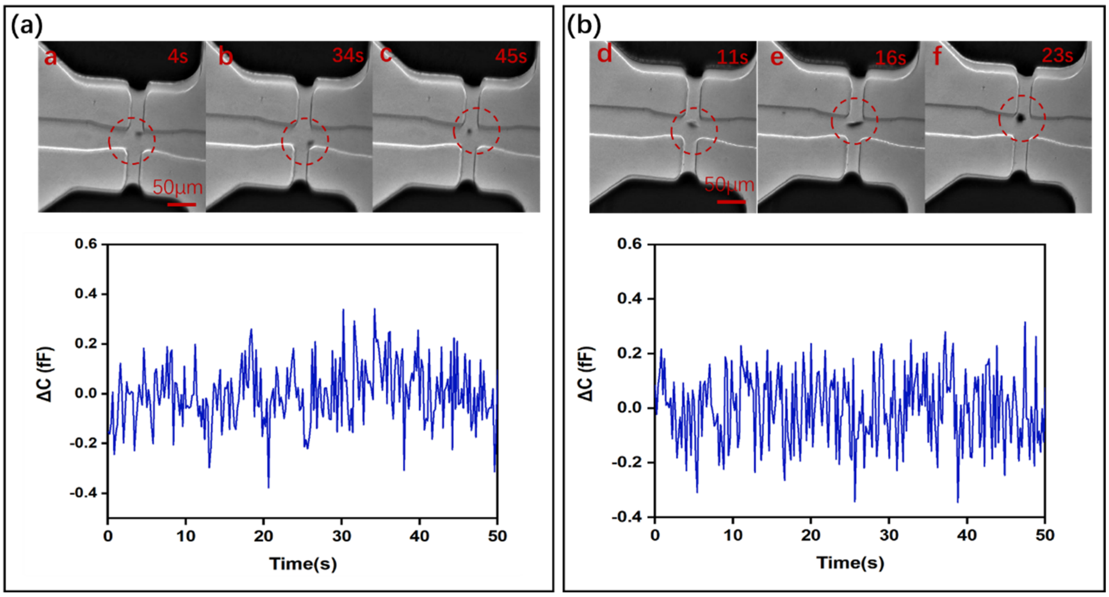

Figure S4. The optical and capacitance counting results of the non-electroplated microelectrodes. (**a**)Tiny particles (< 10 μm) passed through the microelectrodes at 4 s, 34 s, and 45 s with no signal being found. (**b**) Bigger particles (10~15 μm) passed through the microelectrodes at 11 s, 36 s, and 23 s with no signal being found.
